# Robust Deep Learning–based Segmentation of Glioblastoma on Routine Clinical MRI Scans Using Sparsified Training

**DOI:** 10.1148/ryai.2020190103

**Published:** 2020-09-30

**Authors:** Roelant S. Eijgelaar, Martin Visser, Domenique M. J. Müller, Frederik Barkhof, Hugo Vrenken, Marcel van Herk, Lorenzo Bello, Marco Conti Nibali, Marco Rossi, Tommaso Sciortino, Mitchel S. Berger, Shawn Hervey-Jumper, Barbara Kiesel, Georg Widhalm, Julia Furtner, Pierre A. J. T. Robe, Emmanuel Mandonnet, Philip C. De Witt Hamer, Jan C. de Munck, Marnix G. Witte

**Affiliations:** From the Department of Radiation Oncology, The Netherlands Cancer Institute, Plesmanlaan 121, 1066 CX Amsterdam, the Netherlands (R.S.E., M.v.H., M.G.W.); Department of Radiology and Nuclear Medicine, Amsterdam UMC, Location Vrije Universiteit Amsterdam, Amsterdam, the Netherlands (M.V., F.B., H.V., J.C.d.M.); Neurosurgical Center Amsterdam, Amsterdam UMC, Location Vrije Universiteit Amsterdam, Amsterdam, the Netherlands (D.M.J.M., P.C.D.W.H.); Institutes of Neurology & Healthcare Engineering, University College London, London, England (F.B.); Faculty of Biology, Medicine & Health, Division of Cancer Sciences, University of Manchester and Christie NHS Trust, Manchester, England (M.v.H.); Neurosurgical Oncology Unit, Department of Oncology and Hemato-Oncology, Università degli Studi di Milano, Humanitas Research Hospital, IRCCS, Milan, Italy (L.B., M.C.N., M.R., T.S.); Department of Neurologic Surgery, University of California–San Francisco, San Francisco, Calif (M.S.B., S.H.J.); Department of Neurosurgery, Medical University Vienna, Vienna, Austria (B.K., G.W.); Department of Biomedical Imaging and Image-guided Therapy, Medical University Vienna, Vienna, Austria (J.F.); Department of Neurology & Neurosurgery, University Medical Center Utrecht, Utrecht, the Netherlands (P.A.J.T.R.); and Department of Neurologic Surgery, Hôpital Lariboisière, Paris, France (E.M.).

## Abstract

**Purpose:**

To improve the robustness of deep learning–based glioblastoma segmentation in a clinical setting with sparsified datasets.

**Materials and Methods:**

In this retrospective study, preoperative T1-weighted, T2-weighted, T2-weighted fluid-attenuated inversion recovery, and postcontrast T1-weighted MRI from 117 patients (median age, 64 years; interquartile range [IQR], 55–73 years; 76 men) included within the Multimodal Brain Tumor Image Segmentation (BraTS) dataset plus a clinical dataset (2012–2013) with similar imaging modalities of 634 patients (median age, 59 years; IQR, 49–69 years; 382 men) with glioblastoma from six hospitals were used. Expert tumor delineations on the postcontrast images were available, but for various clinical datasets, one or more sequences were missing. The convolutional neural network, DeepMedic, was trained on combinations of complete and incomplete data with and without site-specific data. Sparsified training was introduced, which randomly simulated missing sequences during training. The effects of sparsified training and center-specific training were tested using Wilcoxon signed rank tests for paired measurements.

**Results:**

A model trained exclusively on BraTS data reached a median Dice score of 0.81 for segmentation on BraTS test data but only 0.49 on the clinical data. Sparsified training improved performance (adjusted *P* < .05), even when excluding test data with missing sequences, to median Dice score of 0.67. Inclusion of site-specific data during sparsified training led to higher model performance Dice scores greater than 0.8, on par with a model based on all complete and incomplete data. For the model using BraTS and clinical training data, inclusion of site-specific data or sparsified training was of no consequence.

**Conclusion:**

Accurate and automatic segmentation of glioblastoma on clinical scans is feasible using a model based on large, heterogeneous, and partially incomplete datasets. Sparsified training may boost the performance of a smaller model based on public and site-specific data.

*Supplemental material is available for this article.*

Published under a CC BY 4.0 license.

**Summary**

Robust deep learning–based segmentation of glioblastoma on routine clinical data can be achieved using a large heterogeneous training dataset or using sparsified training on a combination of public and site-specific data.

**Key Points**

■ Institutional variations in MRI acquisition protocols, hardware, and software result in heterogeneous clinical image datasets that may lack specific sequences; the variability of input data may affect the training and performance of deep learning algorithms.
■ Sparsified training in a dataset consisting of glioblastoma MRI scans improved the performance of a model based on public data to the level of performance of inclusion of center-specific training data, as well as reduced the influence of missing sequences.
■ Models trained on large heterogeneous datasets with missing sequences did not require sparsified training or site-specific training data.

## Introduction

Glioblastoma is the most common form of primary brain tumor in adults (1,2). Radiologic tumor evaluation is typically performed using CT or MRI with two-dimensional measures (3), but with advances in imaging and the need for more detailed tumor quantification, three-dimensional volumetric segmentation in MRI is recommended and is becoming more commonplace (4,5). Preoperative glioma segmentation at MRI can aid localized treatment planning and assessment of quality of care (6,7). Several MRI sequences are acquired to assess tumor location, composition, and extent (8); however, manual tumor segmentation is a time-consuming process and subject to interrater variability (9).

Automated tumor segmentation is an active field of research. The 2017 Multimodal Brain Tumor Segmentation (BraTS) (10) challenge showed promising results with Dice scores of approximately 0.85 for the preoperative tumor core mostly using deep learning–based approaches (11). The NiftyNet (12) project offers standardized tools for deep learning–based automatic segmentation. However, methods developed using high-quality homogeneous and complete research data may suffer from overfitting (13) and fail to achieve high segmentation quality in clinical scans with variable image quality and varying completeness of image sequences. Implementation of automatic segmentation in clinical practice is therefore still lacking.

Heterogeneity of the BraTS data has increased since 2017 by addition of glioblastoma data from eight institutions in The Cancer Imaging Archive (TCIA) (13) and manual ground truth segmentations (14). Previously proposed methods to address the issue of missing sequences generally focused on data imputation (15–17) or network adjustments (16–18), but such approaches have limited generalizability.

In this study, we determined the performance of the automatic glioblastoma segmentation tool DeepMedic (19,20) when using partially incomplete multi-institution clinical imaging data with a wide variability in imaging parameters. We investigated the effects on segmentation performance of center-specific training, missing sequences, and expanding the training data with a heterogeneous dataset. Furthermore, we propose a sparsified training protocol randomly nullifying secondary image sequences and show how this improves robustness in the case of missing data.

## Materials and Methods

Approval of this retrospective study protocol was obtained from institutional review boards, and informed consent from patients was obtained according to local regulations. The data were obtained and anonymized in accordance with the General Data Protection Regulation and Health Insurance Portability and Accountability Act.

### Public BraTS Patient Dataset

From the BraTS dataset updated in 2013 (7) and selected scans from the TCIA (11,12), preoperative baseline scans were selected, resulting in a dataset of 117 patients (median age, 64 years; interquartile range [IQR], 55–73 years; 41 women, 76 men) with four MRI types present: pre- and postcontrast T1-weighted, T2-weighted, and T2-weighted fluid-attenuated inversion recovery (FLAIR). Most scans were obtained at a field strength of 1.5 T (*n* = 59); the remaining used a 3-T scanner (*n* = 35). The segmented tumor core, defined as the union of enhancing and nonenhancing tumor, and necrotic regions were used for analysis. For BraTS, the tumors were manually segmented; for TCIA, they were semiautomatically segmented using GLISTRBoost (*https://www.med.upenn.edu/sbia/glistrboost.html*) and manually corrected.

### Clinical Patient Dataset

Preoperative MR images of 634 adult patients (median age, 59 years; IQR, 49–69 years; 382 men, 244 women, eight unknown) with a histopathologic diagnosis of glioblastoma were collected (Table 1). These patients received consecutive surgical treatment between 2012 and 2013 at one of six multinational tertiary referral hospitals (referred to as hospitals 1–6). Patients either underwent a resection or a biopsy. All patients with at least a preoperative postcontrast T1-weighted scan were included, and no patients were excluded. These data were collected as part of the PICTURE project (*https://www.pictureproject.nl*) (7,9,21–23). The histopathologic diagnosis was determined according to the World Health Organization 2007 criteria (24). Scans were obtained using 21 different MRI scanner models, and most scans were obtained at a field strength of 1.5 T (*n* = 316) or 3 T (*n* = 292), with smaller numbers at 1 T (*n* = 22) or 0.4 T (*n* = 1). Scan resolutions varied, ranging between 0.5- and 6-mm slice thickness. Images had varying MRI parameters because of hospital-specific settings (eg, field of view) and scanner specifications (see Tables E1 and E2 [supplement] for more details). In 54% (345 of 634) of patients, one or more of the secondary sequences were missing (see Table 2).

**Table 1:**
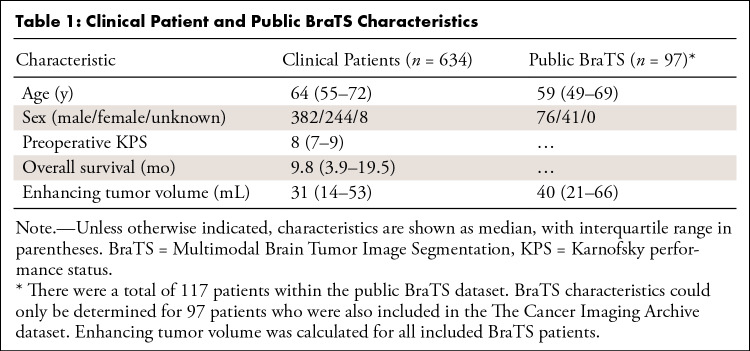
Clinical Patient and Public BraTS Characteristics

**Table 2:**
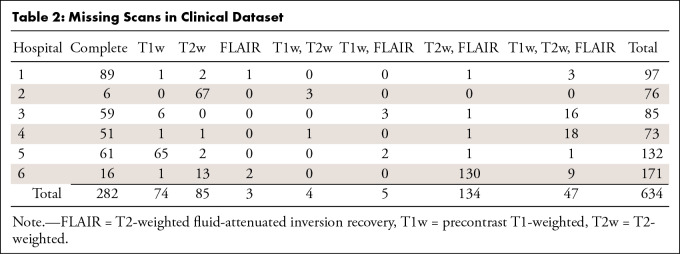
Missing Scans in Clinical Dataset

Tumor segmentation following the Visually AccesSAble Rembrandt Images criteria (25) was performed by a single manual rater with 3 years of experience in neurosurgical residency (D.M.J.M.) under supervision of a neurosurgeon (P.C.D.W.H.) and neuroradiologist (F.B.) using the semiautomatic SmartBrush tool (BrainLab, Feldkirchen, Germany). Performance on preoperative glioblastoma segmentation of this rater was comparable with expert level (9). Tumor volume was defined as the union of the enhancing tumor and enclosed necrosis, which is comparable with tumor core segmentations from BraTS and TCIA glioblastoma with the exclusion of nonenhancing tumor.

Secondary sequences (T1-weighted, T2-weighted, and FLAIR) were rigidly registered to the postcontrast T1-weighted sequences and then rigidly registered to the MNI09a template (*http://nist.mni.mcgill.ca/?p=904*) and thus resampled to 1-mm isotropic voxels. Registrations were performed with the Advanced Normalization Tools (26). Bias field corrections were performed with N4 bias correction (27), and skull stripping was performed with a routine that relied on the Atropos (28) tool.

### Automatic Tumor Segmentation

Automatic segmentations were performed using the convolutional neural network DeepMedic (19,20) as implemented in the NiftyNet (12) framework. DeepMedic consists of two pathways that are 11 layers deep and accepts three-dimensional patches as inputs. The patch size was set to 57 × 57 × 57 voxels with a downsample factor of three, resulting in an output of 9 × 9 × 9 voxels. Training minimized a Dice loss function (29) for 30 000 iterations by using the Adam (30) optimizer. The learning rate started at 0.001 and was divided by 2 every 5000 iterations up to iteration 15 000 and every 1500 iterations thereafter. The last iteration was used for inference on the test datasets. All models were trained and evaluated on a computer equipped with a single Tesla P100 GPU (3584 CUDA cores, 12 GB).

### Sparsified Training

Where scans were missing, empty (zero-filled) scans with the same resolution and orientation as the other scans were inserted. Sparsified training was implemented as an augmentation layer by randomly setting secondary sequences to zero with independent probabilities of 20%. This percentage approximated the frequency of missing sequences in the clinical dataset. The NiftyNet pipeline includes histogram normalization and whitening (12,31). These layers were adjusted to ensure that zero-filled volumes representing missing sequences were unaffected. Consequently, missing sequences in the original data and from the sparsified training augmentation layer were fed to the network as all zero matrices, which, as a result of the whitening layer, corresponds to the mean intensity of the available data.

### Tumor Segmentation Evaluation

Tumor segmentations were evaluated using Dice score, Hausdorff distance, and sensitivity metrics (32). Dice score is defined as:,
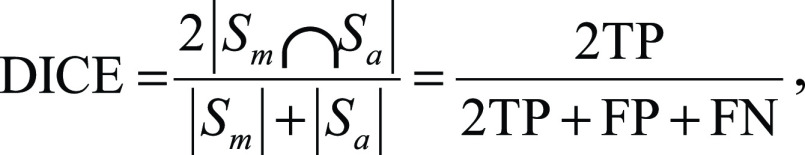
where *S*_*m*_ is the manually segmented tumor voxels, *S*_*a*_ is the automatically segmented tumor voxels, TP is the number of true-positive voxels, FP is the false-positive voxels, and FN is the false-negative voxels. Sensitivity follows as:.
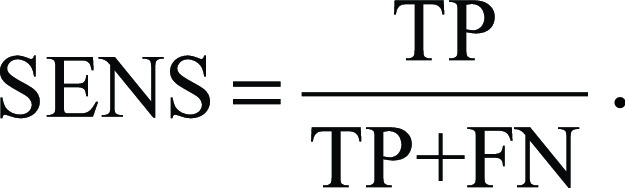


The Hausdorff distance measures the maximum distance between borders and is therefore sensitive to evaluation direction and outliers. We therefore used the undirected 95th percentile Hausdorff distance:,
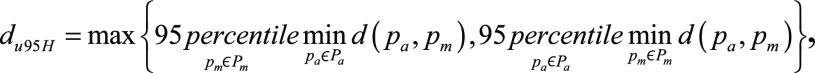
where *P*_*m*_ is the set of vertices describing the border of the manual segmentation, *P*_*a*_ is the set of vertices describing the border of the automatic segmentation, and *d* (*p*_*a*,_ *p*_*m*_) is the distance between two vertices.

### Experimental Design

The BraTS dataset was randomly split into training (80%, *n* = 93), validation (5%, *n* = 6), and testing (15%, *n* = 18) sets. The clinical test set included 20 manually selected patients from hospital 1 with complete imaging for whom the interrater agreement of manual segmentations had been previously studied (*https://doi.org/10.17026/dans-zg9-nhrj*) (9). For hospitals 2 to 6, patients were randomly subdivided into training (70%, *n*_total_ = 350), validation (5%, *n*_total_ = 39), and testing (25%, *n*_total_ = 148) where *n*_total_ is the total number of patients for hospitals 2 to 6. Combinations of training data from different datasets were used to train various models. Validation data were used to confirm convergence, and hyperparameters were then kept constant for all models. Test data were used to evaluate the performance of the different models.

**Figure 1:**
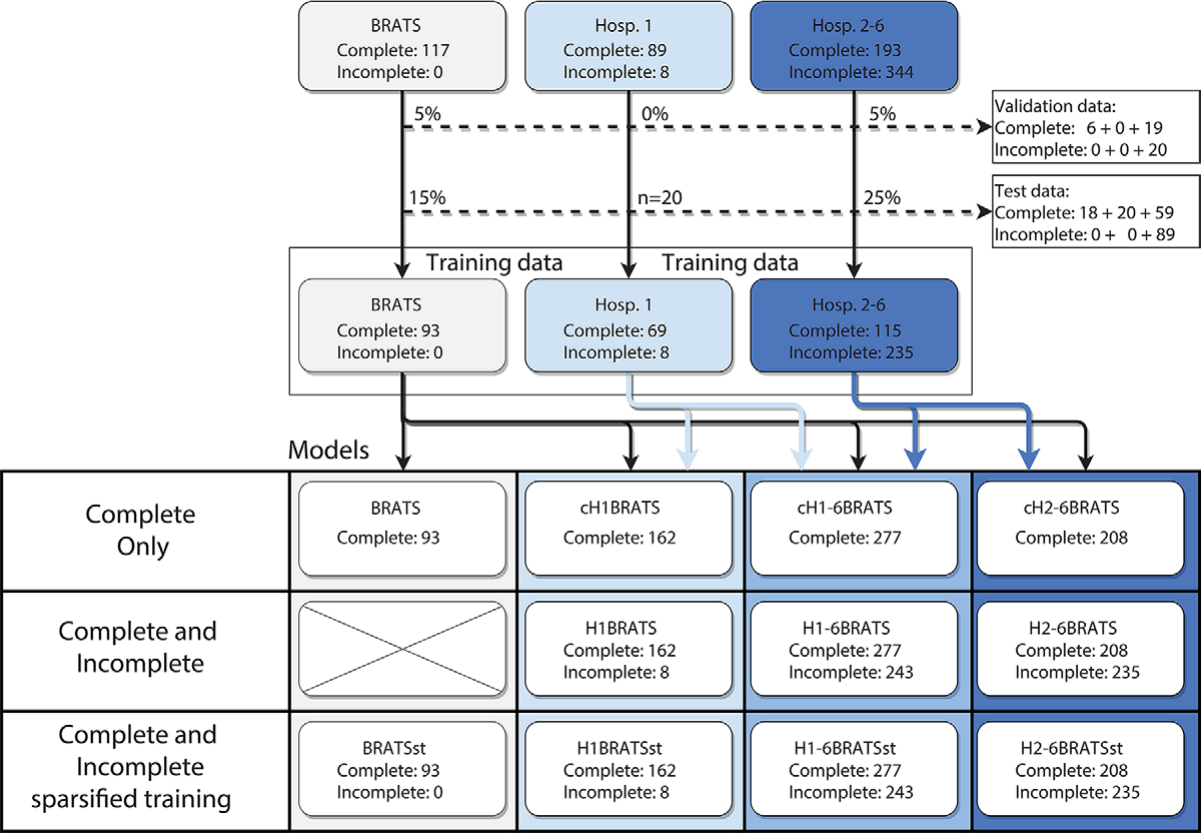
The clinical and public data were divided into three main groups: Multimodal Brain Tumor Image Segmentation (BraTS), hospital 1, and hospitals 2 to 6. The dashed arrows show the (fraction of) patients assigned to the validation and test data. The remaining scans were used to train a total of 11 distinct networks by varying all of the included training data, use of all available data, or only patients with complete imaging, and use of sparsified training. Each model is described by the abbreviation of the included data, preceded with a “c” if only patients with complete imaging were included, and followed by “st” if sparsified training was enabled. The 20 test patients from hospital 1 were previously studied in Visser et al (9).

A total of 11 networks were trained by varying the included hospitals, the inclusion of patients with incomplete imaging, and the use of sparsified training. BraTS patients were included in all models. Figure 1 explains the naming convention for the different models.

The performance on clinical data of a network based on publicly available BraTS data was evaluated. These results show the combined effects of imaging heterogeneity, missing scans, and other issues related to overfitting and domain adaptation. The effects of missing MRI sequences were further investigated by simulating all possible combinations of missing secondary sequences in the test dataset of 20 patients. Sparsified training was explored as a strategy to mitigate the impact of missing sequences. The impact of image heterogeneity between institutes was estimated by evaluating networks trained with and without hospital 1 data on the set of 20 patients, thus assessing the need for hospital-specific data in the training set.

### Statistical Analyses

Dice scores comparing automatic to manual segmentations of all model pairs with and without sparsified training, as well as all model pairs with and without hospital 1–specific training data, were compared using Wilcoxon signed rank tests for paired measurements (using R, version 3.6.1, *https://www.r-project.org/*) (33) for complete imaging and all combinations of simulated missing scans in the set of 20 patients from hospital 1. A conservative multiple testing correction was applied, calculating adjusted *P* values using a single Bonferroni correction on all (*n* = 72) calculated *P* values. Adjusted *P* values less than .05 were considered significant.

## Results

### Segmentation Performance on the Clinical Patient Dataset

**Figure 2:**
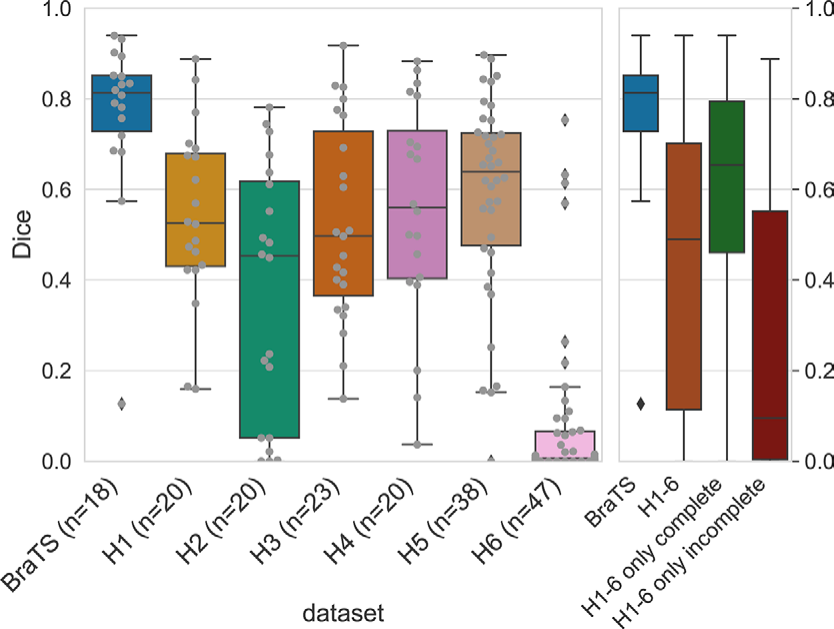
Dice scores of a model trained on publicly available Multimodal Brain Tumor Image Segmentation (BraTS) data, evaluated on a retrospective test cohort from six hospitals and BraTS. Gray bullets indicate individual scans. The right panel shows both the pooled results for hospitals 1 to 6 and the subsets of patients with complete and incomplete data.

The BraTS trained model achieved a median Dice score of 0.81 on BraTS test data, which was comparable with scores cited in the literature (11,20,34). Performance on test datasets of the clinical data (hospitals 1–6) was substantially lower, with an overall median Dice score of 0.49 (Fig 2). Dice scores were similar (medians around 0.55) between hospitals 1, 3, 4, and 5 but highly variable in hospital 2 and very low (median: 0.01) in hospital 6. Results for the 95th percentile Hausdorff distance and sensitivity can be found in Figure E1 (supplement).

### Segmentation Performance with Missing MRI Sequences

**Figure 3:**
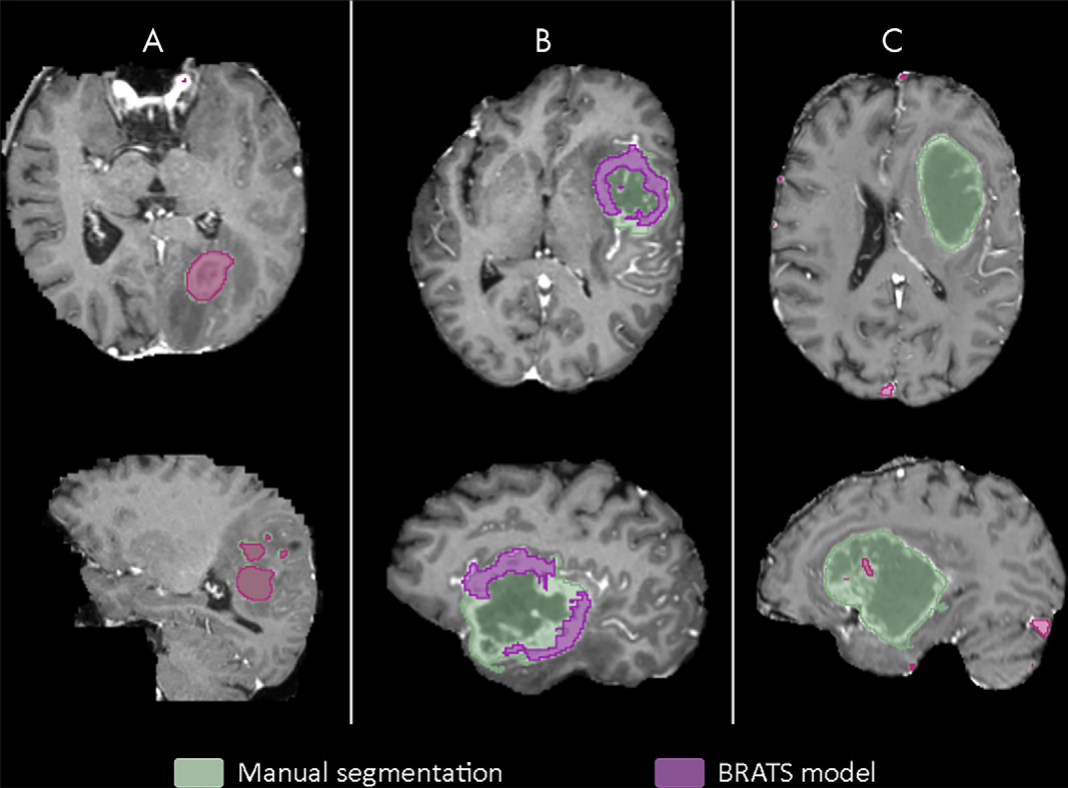
Segmentations generated by the Multimodal Brain Tumor Image Segmentation (BraTS) model overlaid on the manual segmentations for, *A*, BraTS test patient (Dice score, 0.85), *B*, clinical patient with complete secondary imaging (Dice score, 0.43), and, *C*, patient with missing T2-weighted and T2-weighted fluid-attenuated inversion recovery images (Dice score, 0.001).

In hospital 2 the T2-weighted sequences were missing, and in hospital 6, both the T2-weighted and FLAIR sequences for a large fraction of patients were missing. The subset of all test patients missing one or more sequences had a median Dice score of 0.095 (IQR, 0.005–0.55), and for the patients with all sequences available, this was 0.65 (IQR, 0.46–0.79). Sensitivity and *d*_u95H_ are shown in Figure E2 (supplement). Figure 3 shows examples of the segmentation result of the BraTS model for a BraTS test patient, a clinical patient with all secondary sequences available, and a clinical patient who had missing T2-weighted and FLAIR sequences.

**Figure 4:**
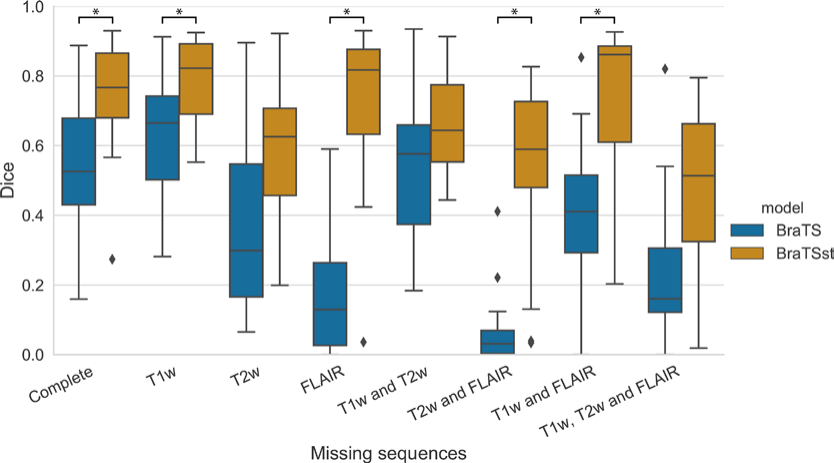
Dice performances of the Multimodal Brain Tumor Image Segmentation (BraTS) model with sparsified training (orange) and without sparsified training (blue). Missing data were simulated by artificially setting secondary images to zero during inference for the 20 test patients from hospital 1. Significant differences (adjusted *P* < .05) are indicated by an asterisk. FLAIR = fluid-attenuated inversion recovery, T1w = T1-weighted, T2w = T2-weighted.

In Figure 4, the effect of a particular missing sequence, or combination of missing sequences, is shown for the BraTS model using the 20 test patients from hospital 1. Although a missing precontrast T1-weighted sequence did not reduce performance (median Dice score: 0.66), missing T2-weighted (0.30) and especially missing FLAIR (0.13) sequences led to lower median Dice scores. A number of interactions can be observed. For example, missing precontrast T1-weighted and FLAIR (0.41) outperforms missing FLAIR (0.13), and no secondary sequences (0.16) appears better than to use precontrast T1-weighted only (0.03). This same trend was observed in the other institutes (Figure E3 [supplement]).

Sparsified training in the BraTS_st_ model substantially improved performance for all combinations of missing sequences (Figure 4), with significant adjusted *P* values for missing FLAIR and/or precontrast T1-weighted sequences, as well as missing FLAIR and T2-weighted (adjusted *P* values of all model comparisons can be found in Tables E3 and E4 [supplement]). An especially large improvement was seen in the case of missing FLAIR. Also, complete test data of the BraTS_st_ model (median Dice score: 0.77) had a higher score than that of the BraTS model (0.53), although it was statistically nonsignificant (adjusted *P* = .095). Results showed to be robust to the level of sparsity (see Figure E4 [supplement]).

### Image Heterogeneity in Training Sets Improved Segmentation

**Figure 5:**
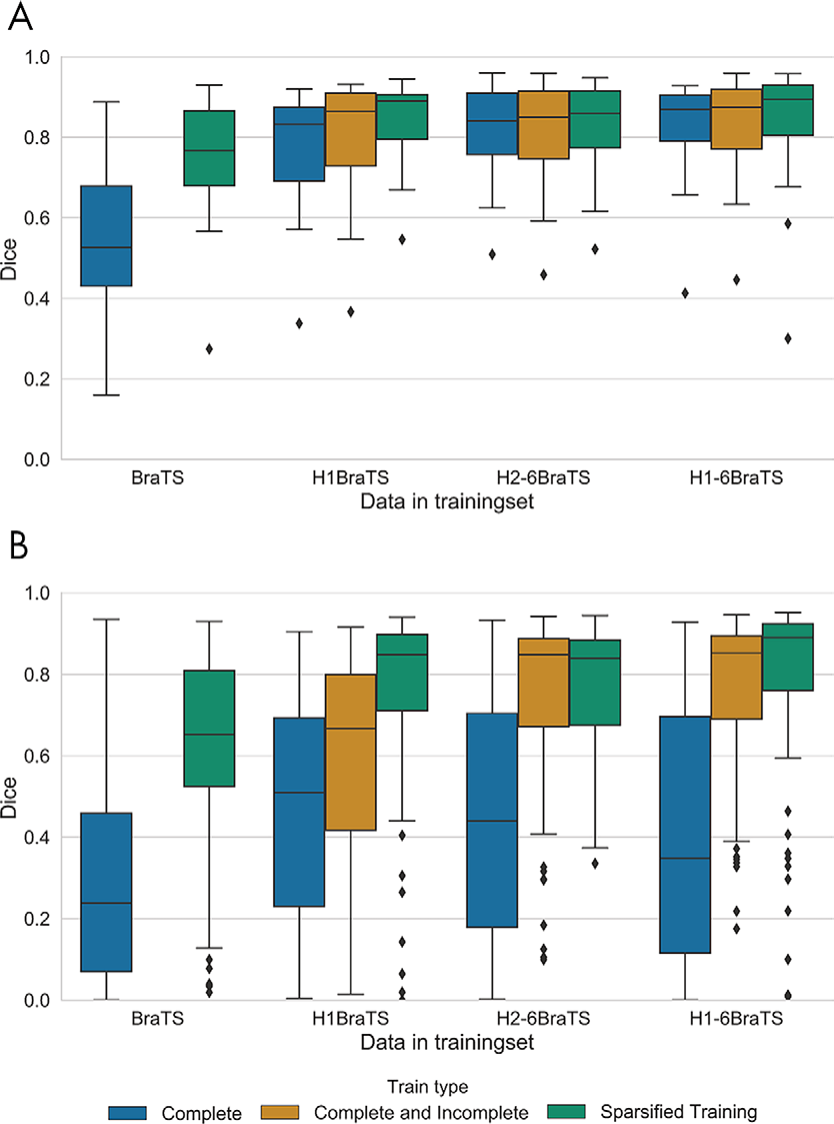
Median Dice scores of all models in Figure 1 on, *A*, 20 test patients from hospital 1 with complete imaging and, *B*, for all combinations of simulated missing scans. BraTS = Multimodal Brain Tumor Image Segmentation.

Evaluation results for models trained with the inclusion of clinical data are shown in Figure 5. When performing inference using all available scans of the 20 patients who were left out of hospital 1 (Fig 5, *A*), each model incorporating clinical data outperformed the BraTS model.

Omission of patients from hospital 1 during training did not significantly reduce the performance relative to the corresponding models using all available patients, unlike the models only trained on BraTS data (adjusted *P* < .05 for complete imaging [median Dice score difference: −0.07], only postcontrast T1-weighted available [−0.57], missing FLAIR [−0.50], and missing T2-weighted [−0.35]). Sparsified training neither significantly improved nor reduced the performance of models that included data from hospitals 2 to 6 (H_2–6_BraTS and H_1–6_BraTS). See Tables E3 and E4 (supplement) for tables with more detailed results, including median differences, 95% confidence intervals, and nonadjusted *P* values.

**Figure 6:**
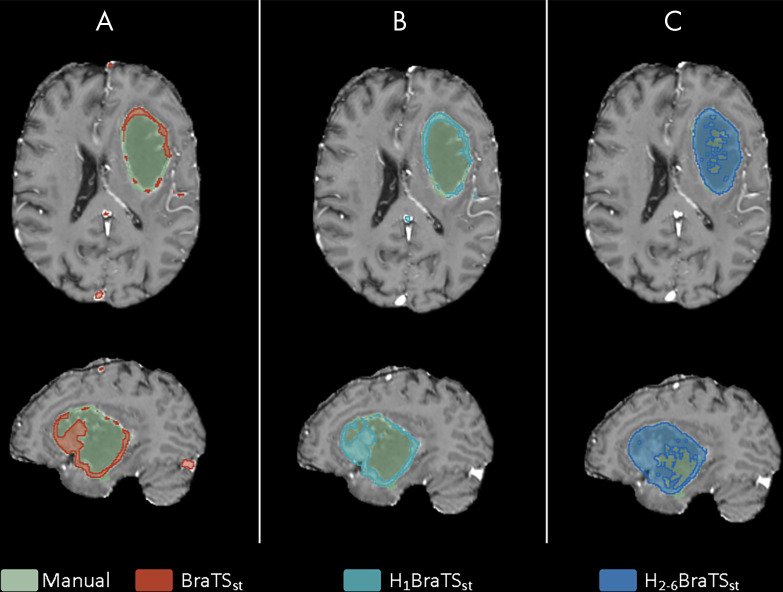
Segmentation results for the, *A*, BraTSst (Dice score, 0.41), *B*, H_1_BraTSst (Dice score, 0.52), and, *C*, H_2–6_BraTSst (Dice score, 0.82) models for the patient with missing T2-weighted and T2-weighted fluid-attenuated inversion recovery from Figure 3, *C*. BraTS = Multimodal Brain Tumor Image Segmentation.

For simulated missing sequences (Fig 5, *B*), Dice scores for models trained exclusively on complete image sets were reduced. The inclusion of patients with incomplete imaging during training restored the performance for the H_1–6_BraTS and H_2–6_BraTS models, but only partially for the H_1_BraTS model (hospital 1 had only eight patients with incomplete imaging). Sparsified training restored the performance of the H_1_BraTS_st_ model to the same level of performance (median Dice scores ≥ 0.84) as the models including the other hospitals. Omission of institution-specific data from hospital 1 did not significantly reduce performance for the H_1–6_BraTS_st_ model (adjusted *P* ≥ .08). Figure 6 shows the segmentation results of the BraTS_st_, H_1_BraTS_st_, and H_2–6_BraTS_st_ models for the incomplete test patient of Figure 3. Performance (Dice, sensitivity, and *d*_*u*95*H*_) of the H_2–6_BraTS_st_ model is shown in more detail for individual test datasets in Figure E1 (supplement).

## Discussion

We have shown the feasibility of obtaining accurate automatic glioblastoma segmentations using deep learning without the need for hospital-specific training data, even in the case of missing secondary sequences. In clinical practice, missing sequences is a likely occurrence; for a small majority of clinical scans collected for this study, one or more of the secondary sequences were missing. A sparsified training strategy improved a model based on public data for use both on complete and incomplete clinical datasets and was able to bring a model based on single-institute data (plus those publicly available) to the level of a much larger multi-institute model.

We have shown that the models trained with the largest dataset (eg, H_2–6_BraTS_st_) reach median Dice scores (approximately 0.85 both on complete and incomplete data) that are comparable with the top-performing algorithms from the BraTS challenge and the results in Perkuhn et al (35), yet still below the excellent interrater agreement of 0.94 in the BraTS data (10) and of 0.93 in a subset of the dataset described in this article (9).

In this study, we have used the DeepMedic implementation in NiftyNet. We have chosen to use DeepMedic because it was shown to be one of the top performers of the BraTS 2016 challenge, and several studies have reported Dice scores of DeepMedic (eg, as reference value) trained and evaluated on the BraTS data (mix of various gliomas). Results ranged between 0.72 and 0.83 (20), and submissions were from the BraTS proceedings (11,34).

Incomplete imaging was shown to greatly reduce performance in the unadjusted DeepMedic network. Even though introducing sparsified training and clinical data to the training dataset improved segmentation performance for incomplete data, the best performance was achieved if all sequences were available. Systematic use of all sequences in clinical practice is preferred (as advocated in Freyschlag et al [36]) and would help overcome this issue. Despite the efforts toward standardization, robustness to missing sequences remains valuable for analyzing real-world cohorts, flexibility to changes to standardized protocols, and bringing the benefits of automated segmentation to as many patients as possible.

The largest performance improvement that resulted from introducing sparsified training to the BraTS model was observed with missing FLAIR images, indicating that the information used by the model from the FLAIR could also be extracted from a combination of other sequences. Next to the variations in imaging protocols, variations in preprocessing (ie, the registration algorithm, atlas, and skull stripping algorithm) may have contributed to the heterogeneity between the public patient data and the clinical dataset. The largest performance differences could, however, be attributed to missing sequences. The performance improvement from sparsified training in the BRaTS model for complete test data might be attributed to a general regularizing effect of sparsified training, reducing overfitting.

The DeepMedic implementation in NiftyNet (12) has minor differences compared with the original implementation (20), which used the now-deprecated Theano backend. However, we expect that these differences have a relatively minor impact on performance. The NiftyNet implementation does not include dropout in the final fully connected layers and uses a Dice-based loss function and Adam as an optimizer as opposed to RMSProp with Nesterov momentum in the original DeepMedic implementation. NiftyNet allows various datasets and augmentation options to be easily combined, and it facilitates publication of trained models (*https://niftynet.readthedocs.io/en/dev/model_zoo.html*).

Results of this study focused on the test data of hospital 1. A “leave one hospital out” cross-validation could provide more detailed results but was practically infeasible because of the computational costs involved.

The high *d*_*u*95*H*_ (Figure E2 [supplement]) for some patients indicated that, for these patients, some false-positive regions were found relatively distant to the boundary of the tumor. This may limit the usability of these models for some applications. Improvements could be made by further postprocessing, inclusion of a distance component to the loss-function of the convolutional neural network, or further hyperparameter tuning (eg, sparsity frequency, numbers of layers and feature maps, number of iterations or learning rate). Improvements using a fully connected conditional random field (37) or the use of ensembles (38) were previously explored but were left out in our analyses because of the added complexity and training and inference times.

Our results showed that introducing sparsified training and a large heterogeneous training dataset improved the robustness of automatic segmentation of glioblastoma on routine clinical MRI. Improved robustness of automatic segmentation will bring the application of these algorithms a step closer to clinical practice.

## SUPPLEMENTAL TABLES

Tables E1–E4 (PDF)

## SUPPLEMENTAL FIGURES

Figure E1:

Figure E2:

Figure E3:

Figure E4:
