# Supplementary material for: Robust Deep Learning–based Segmentation of Glioblastoma on Routine Clinical MRI Scans Using Sparsified Training
Source: Radiol Artif Intell. 2020 Sep 30;2(5):e190103. doi: 10.1148/ryai.2020190103 (PMC8082349; doi:10.1148/ryai.2020190103)
Supplement: Tables E1–E4 (PDF) [file ryai190103suppa1.pdf]

©RSNA, 2020
10.1148/ryai.2020190103

Table E1. MR Scanner Characteristics

Vendor and Model	Clinical patients (*n* = 634)	Public BraTS (*n* = 117)
GE Medical Systems		
Discovery MR750	116	0
Signa Excite	71	14
Signa HDxt	25	0
Signa HDx	2	0
Genesis signa	0	24
Phillips Medical Systems		
Achieva	96	9
Intera	60	2
Panorama HFO	17	0
Ingenia	8	0
Ingenuity	6	0
NT Intera	3	0
Gyroscan NT	2	0
Siemens		
Sonata	21	0
TrioTim	66	2
Verio	77	4
Aera	2	0
Avanto	52	5
Espree	4	0
Magnetom Essenza	2	0
Skyra	1	0
Magnetom Vision	0	6
Symphony	0	3
Trio	0	1
Toshiba		
Titan 3T	2	0
Hitachi Medical Corporation		
Aperto	1	0

Note.—The field strengths for the clinical patient dataset were 3 T (*n* = 293), 1.5 T (*n* = 318), 1 T (*n* = 22), and 0.4 T (*n* = 1). The field strengths for the (Multimodal Brain Tumor Image Segmentation) BraTS dataset were 3 T (*n* = 35) and 1.5 T (*n* = 59). BraTS characteristics could only be determined for 97 patients that were also included in the TCGA dataset. From this dataset the scanner vendor and model could not be extracted from the DICOM tags for 28 patients, and the field strength was unavailable for 3 patients.

Table E2. MR Scanner Parameters

Scan Type	Voxel dimensions (mm x mm x mm)			TR	TE	Flip angle
A. Clinical Patients (*n* = 634)						
Postcontrast T1-weighted	1.00 (0.52–1.00)	1.00 (0.52–1.00)	1.00 (1.00–2.00)	1800 (7.29–1800)	2.47 (2.47–6.68)	12 (12–30)
Precontrast T1-weighted	0.98 (0.47–0.96)	0.98 (0.47–0.96)	5.00 (4.00–5.00)	2000 (550–1540)	12 (9–12)	90 (70–90)

T2-weighted	0.53 (0.45–0.53)	0.53 (0.45–0.53)	5.00 (1.00–5.00)	3000 (3000–5305)	115 (89.62–101.7)	90 (90–120)
FLAIR	0.50 (0.47–0.90)	0.50 (0.47–0.90)	1.00 (1.00–4.00)	6050 (6004–8002)	100 (121.8–34.0)	90 (90–120)
B. Public BraTS (*n* = 117)						
Postcontrast T1-weighted	0.86 (0.49–0.94)	0.86 (0.49–0.94)	3 (2.00–5.00)	537 (417–718)	8 (6.4–13)	90 (35–90)
Precontrast T1-weighted	0.86 (0.68–0.94)	0.86 (0.68–0.94)	5 (4.00–5.00)	500 (417–692)	8.1 (6.4–14)	90 (70–90)
T2-weighted	0.78 (0.47–0.94)	0.78 (0.47–0.94)	5 (3.00–5.00)	3500 (3000–4000)	100 (89–105)	90 (90–90)
FLAIR	0.86 (0.62–0.94)	0.86 (0.62–0.94)	5 (3.00–5.00)	10002 (9334–10004)	135 (125–147)	90 (90–90)

Note.—Characteristics are shown as median (interquartile range). Scans were not necessarily obtained in identical slice directions, therefore dimensions here are in the *i*, *j*, *k* coordinates where *k* is the slice direction The inversion time for T2-weighted fluid attenuated inversion recovery (FLAIR) sequences was 25000 (interquartile range [IQR], 1650.00–2313.75) in the clinical patient dataset and 2200 (IQR, 2200–2330) in the Multimodal Brain Tumor Image Segmentation (BraTS) dataset. TE = echo time, TR = repetition time

Table E3: Median differences, 95% confidence intervals, *P* values and Bonferroni adjusted (72 tests) *P* values comparing Dice scores of model pairs with and without sparsified training, determined using Wilcoxon signed rank tests.

**Models:**	**Complete**	**T1w**	**T2w**	**FLAIR**	**T1w-FLAIR**	**T2w-FLAIR**	**T1w-T2w**	**T1w-T2w-FLAIR**
**BraTS-BraTSst**								
Median difference	–0.176	–0.161	–0.193	–0.550	–0.442	–0.555	–0.137	–0.328
Confidence Interval (min)	–0.303	–0.218	–0.337	–0.662	–0.461	–0.609	–0.184	–0.403
Confidence Interval (max)	–0.098	–0.102	–0.128	–0.473	–0.271	–0.361	–0.063	–0.133
P value	0.00132	0.00019	0.00132	0.00009	0.00012	0.00009	0.00250	0.00151
P value (adjusted)	**0.095**	**0.014**	0.095	**0.006**	**0.009**	**0.006**	0.180	0.109
**H1BraTS-H1BraTSst**								
Median difference	–0.019	–0.125	–0.095	–0.083	–0.388	–0.054	–0.201	–0.205
Confidence Interval (min)	–0.053	–0.236	–0.174	–0.139	–0.449	–0.128	–0.281	–0.258
Confidence Interval (max)	–0.006	–0.095	–0.058	–0.057	–0.285	–0.034	–0.134	–0.145
P value	0.01237	0.00009	0.00019	0.00019	0.00009	0.00132	0.00009	0.00010
P value (adjusted)	0.891	**0.006**	**0.014**	**0.014**	**0.006**	0.095	**0.006**	**0.007**
**H2–6BraTS-H2–6BraTSst**								
Median difference	–0.005	0.010	0.008	–0.043	–0.044	0.011	0.009	0.029
Confidence Interval (min)	–0.024	–0.007	–0.006	–0.089	–0.130	–0.014	–0.018	0.009
Confidence Interval (max)	0.011	0.017	0.024	–0.026	–0.018	0.023	0.038	0.058
P value	0.47813	0.20433	0.16718	0.00116	0.00455	0.11689	0.50159	0.01688
P value (adjusted)	1.000	1.000	1.000	0.084	0.328	1.000	1.000	1.000
**^{1}H–6BraTS-^{1}H–6BraTSst**								
Median difference	–0.006	–0.019	–0.001	–0.027	–0.051	–0.017	–0.021	–0.021
Confidence Interval (min)	–0.029	–0.033	–0.011	–0.157	–0.168	–0.033	–0.054	–0.045
Confidence Interval (max)	0.002	0.004	0.015	–0.020	–0.023	–0.004	–0.005	–0.003
P value	0.10046	0.07932	0.88129	0.00078	0.00573	0.02277	0.01688	0.03037
P value (adjusted)	1.000	1.000	1.000	0.056	0.413	1.000	1.000	1.000

Table E4: Median differences, 95% confidence intervals, *P* values and Bonferroni adjusted *P* values comparing Dice scores of model pairs with and without center specific training data, determined using Wilcoxon signed rank tests.

Models:	Complete	T1w	T2w	FLAIR	T1w-FLAIR	T2w-FLAIR	T1w-T2w	T1w-T2w-FLAIR
**BraTS–cH1BraTS**								
Median difference	−0.243	−0.018	−0.346	−0.496	0.084	−0.572	0.142	−0.043
Confidence Interval (min)	−0.366	−0.103	−0.475	−0.589	−0.026	−0.614	0.048	−0.133
Confidence Interval (max)	−0.131	0.126	−0.214	−0.342	0.178	−0.338	0.333	0.095
P value	0.00116	0.76520	0.00068	0.00009	0.17896	0.00009	0.01237	0.41146
P value (adjusted)	0.084	1.000	**0.049**	**0.006**	1.000	**0.006**	0.891*	1.000
**BraTSst–H1BraTSst**								
Median difference	−0.069	−0.029	−0.223	−0.075	−0.025	−0.173	−0.095	−0.186
Confidence Interval (min)	−0.129	−0.074	−0.287	−0.133	−0.067	−0.248	−0.173	−0.249
Confidence Interval (max)	−0.044	−0.006	−0.142	−0.056	0.003	−0.132	−0.058	−0.138
P value	0.00019	0.02063	0.00012	0.00012	0.07314	0.00016	0.00194	0.00010
P value (adjusted)	**0.014**	1.000	**0.009**	**0.009**	1.000	**0.012**	0.140	**0.007**
**cH2–6BraTS–cH1–6BraTS**								
Median difference	−0.017	0.388	−0.014	0.063	0.289	−0.131	0.013	0.029
Confidence Interval (min)	−0.032	0.265	−0.058	0.024	0.220	−0.198	0.007	0.019
Confidence Interval (max)	0.020	0.430	0.002	0.121	0.336	−0.072	0.058	0.063
P value	0.45527	0.00009	0.09296	0.00511	0.00009	0.00052	0.00220	0.00014
P value (adjusted)	1.000	**0.006***	1.000	0.368*	**0.006***	**0.037**	0.159*	**0.010***
**H2–6BraTS–[1]H–6BraTS**								
Median difference	−0.008	−0.006	−0.008	−0.006	−0.003	−0.014	−0.003	−0.012
Confidence Interval (min)	−0.024	−0.021	−0.027	−0.026	−0.028	−0.028	−0.018	−0.028
Confidence Interval (max)	0.002	0.016	−0.005	0.031	0.016	−0.008	0.019	−0.005
P value	0.07932	0.70891	0.00132	0.76520	0.57549	0.00132	0.97022	0.00116
P value (adjusted)	1.000	1.000	0.095	1.000	1.000	0.095	1.000	0.084
**H2–6BraTSst–[1]H–6BraTSst**								
Median difference	−0.015	−0.019	−0.017	−0.017	−0.018	−0.037	−0.035	−0.066
Confidence Interval (min)	−0.030	−0.039	−0.050	−0.034	−0.050	−0.057	−0.079	−0.115
Confidence Interval (max)	−0.005	−0.008	−0.006	−0.004	−0.011	−0.018	−0.019	−0.029
P value	0.01000	0.00642	0.00803	0.02277	0.00642	0.00359	0.00151	0.00511
P value (adjusted)	0.720	0.463	0.578	1.000	0.463	0.259	0.109	0.368

For all *P* values < 1, an asterisk indicates that the median Dice of the model without center-specific data were higher than the model with center-specific data.
