# Supplementary figures and images for: Robust Deep Learning–based Segmentation of Glioblastoma on Routine Clinical MRI Scans Using Sparsified Training
Source: Radiol Artif Intell. 2020 Sep 30;2(5):e190103. doi: 10.1148/ryai.2020190103 (PMC8082349; doi:10.1148/ryai.2020190103)

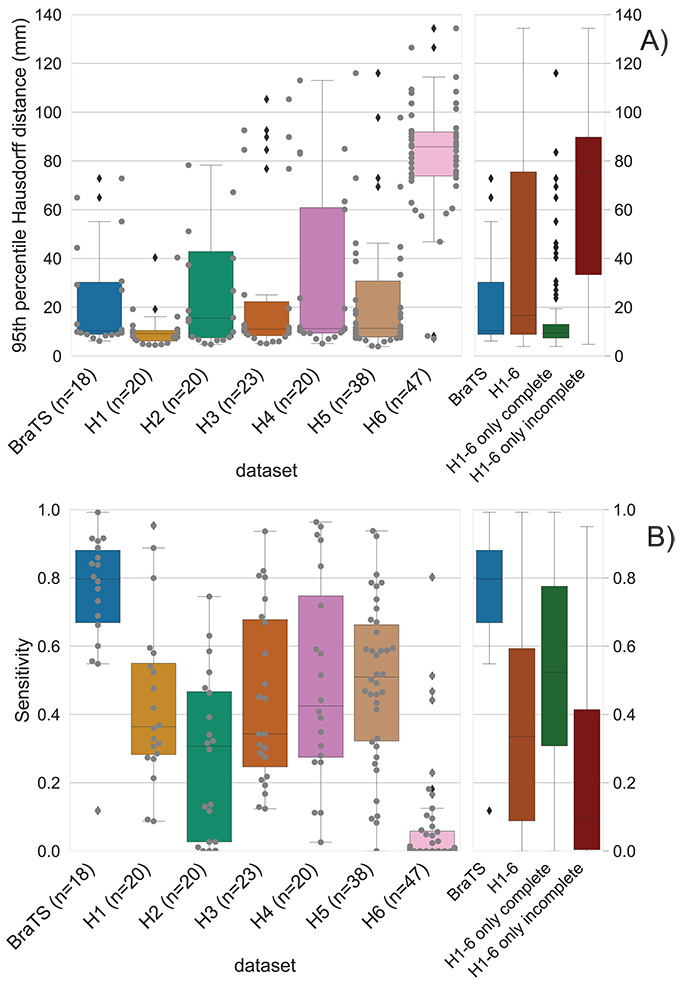

Supplement: Figure E1: [file ryai190103suppf1.jpg]

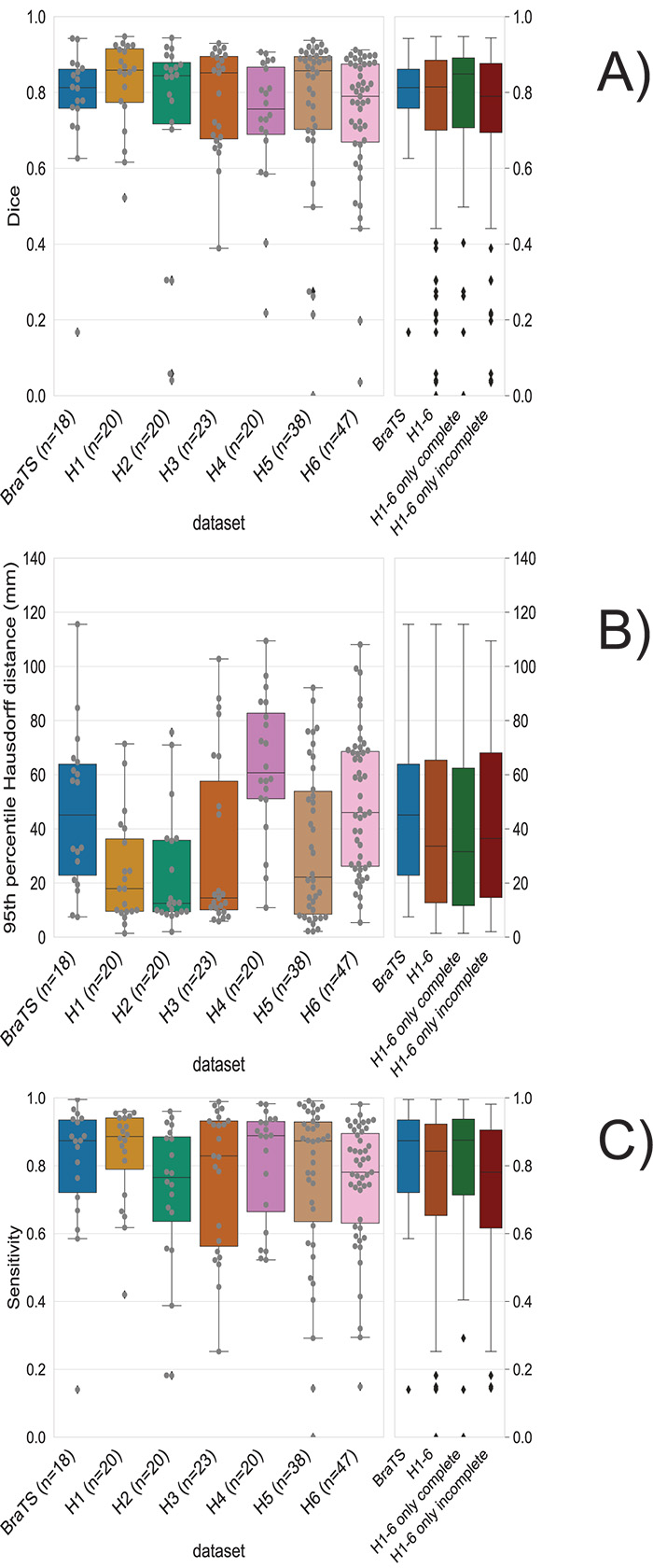

Supplement: Figure E2: [file ryai190103suppf2.jpg]

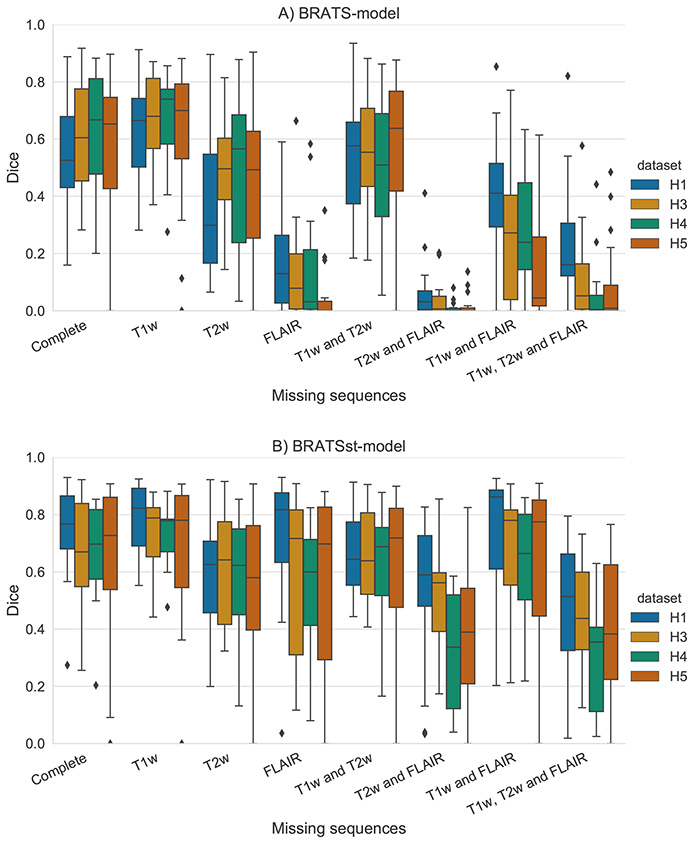

Supplement: Figure E3: [file ryai190103suppf3.jpg]

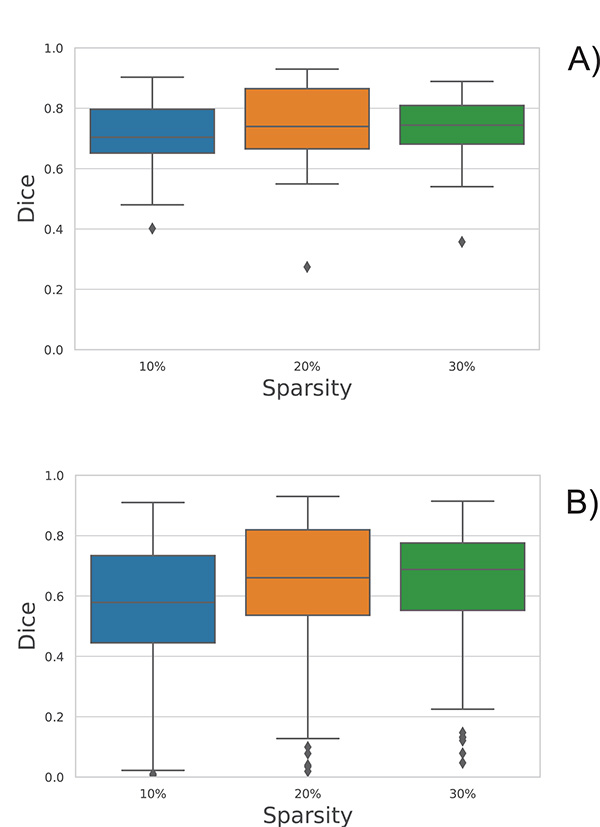

Supplement: Figure E4: [file ryai190103suppf4.jpg]
